# 8,8-Dimethyl-8,9-dihydro-7*H*-chromeno[2,3-*b*]quinoline-10,12-dione

**DOI:** 10.1107/S1600536813001050

**Published:** 2013-01-19

**Authors:** Thothadri Srinivasan, Panneerselvam Yuvaraj, Boreddy S. R. Reddy, Devadasan Velmurugan

**Affiliations:** a Centre of Advanced Study in Crystallography and Biophysics, University of Madras, Guindy Campus, Chennai 600 025, India; b Industrial Chemistry Laboratory, Central Leather Research Institute, Adyar, Chennai 600 020, India

## Abstract

In the title compound, C_18_H_15_NO_3_, the fused benzopyran and pyridine rings are essentially coplanar [r.m.s. deviation = 0.0533 Å with a maximum deviation of 0.080 (1) Å for a benzene C atom]. The cyclo­hexa­none ring adopts an envelope conformation with the dimethyl-substituted C atom 0.660 (2) Å out of the plane formed by the remaining ring atoms (r.m.s. deviation = 0.0305 Å). The dihedral angle between the mean planes of the pyran and cyclo­hexa­none rings is 12.95 (6)°. In the crystal, mol­ecules are linked *via* C—H⋯O hydrogen bonds, leading to chains running along [011].

## Related literature

For the uses and biological importance of diketones, see: Bennett *et al.* (1999 ▶); Sato *et al.* (2008 ▶). For a related structure, see: Öztürk Yildirim *et al.* (2012) ▶.
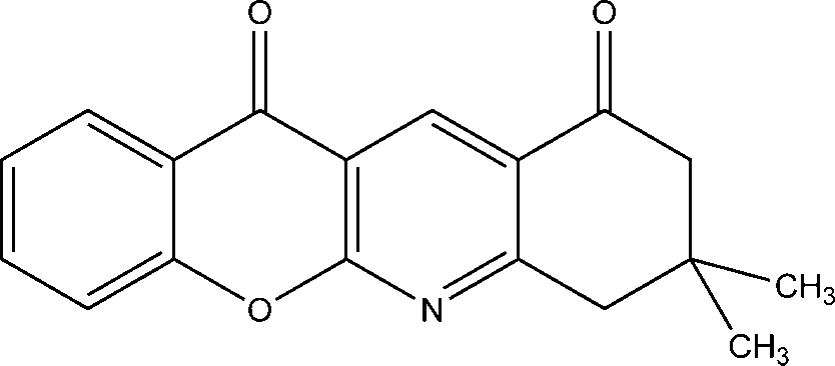


## Experimental

### 

#### Crystal data

C_18_H_15_NO_3_
*M* *_r_* = 293.31
Triclinic, 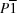

*a* = 7.4426 (6) Å
*b* = 10.5117 (9) Å
*c* = 10.6887 (9) Å
α = 60.939 (4)°
β = 88.107 (5)°
γ = 77.546 (5)°
*V* = 711.22 (10) Å^3^
*Z* = 2
Mo *K*α radiation
μ = 0.09 mm^−1^
*T* = 293 K
0.30 × 0.25 × 0.20 mm

#### Data collection

Bruker SMART APEXII area-detector diffractometer
Absorption correction: multi-scan (*SADABS*; Bruker, 2008 ▶) *T*_min_ = 0.972, *T*_max_ = 0.982
10655 measured reflections
2949 independent reflections
2427 reflections with *I* > 2σ(*I*)
*R*_int_ = 0.031

#### Refinement

*R*[*F* ^2^ > 2σ(*F* ^2^)] = 0.040
*wR*(*F* ^2^) = 0.118
*S* = 1.05
2949 reflections
201 parameters
H-atom parameters constrained
Δρ_max_ = 0.17 e Å^−3^
Δρ_min_ = −0.22 e Å^−3^

### 

Data collection: *APEX2* (Bruker, 2008 ▶); cell refinement: *SAINT* (Bruker, 2008 ▶); data reduction: *SAINT*; program(s) used to solve structure: *SHELXS97* (Sheldrick, 2008 ▶); program(s) used to refine structure: *SHELXL97* (Sheldrick, 2008 ▶); molecular graphics: *ORTEP-3* (Farrugia, 2012 ▶); software used to prepare material for publication: *SHELXL97* and *PLATON* (Spek, 2009 ▶).

## Supplementary Material

Crystal structure: contains datablock(s) global, I. DOI: 10.1107/S1600536813001050/pv2617sup1.cif

Structure factors: contains datablock(s) I. DOI: 10.1107/S1600536813001050/pv2617Isup2.hkl

Supplementary material file. DOI: 10.1107/S1600536813001050/pv2617Isup3.cml

Additional supplementary materials:  crystallographic information; 3D view; checkCIF report

## Figures and Tables

**Table 1:** Hydrogen-bond geometry (Å, °)

*D*—H⋯*A*	*D*—H	H⋯*A*	*D*⋯*A*	*D*—H⋯*A*
C15—H15⋯O2^i^	0.93	2.43	3.250 (2)	147

Symmetry code: (i) 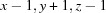.

**Fig. 1.**
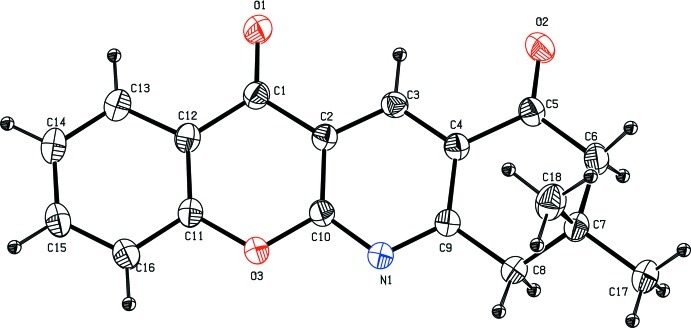
The molecular structure of the title compound, showing displacement ellipsoids drawn at the 30% probability level. H atoms are presented as small spheres of arbitrary radius.

**Fig. 2.**
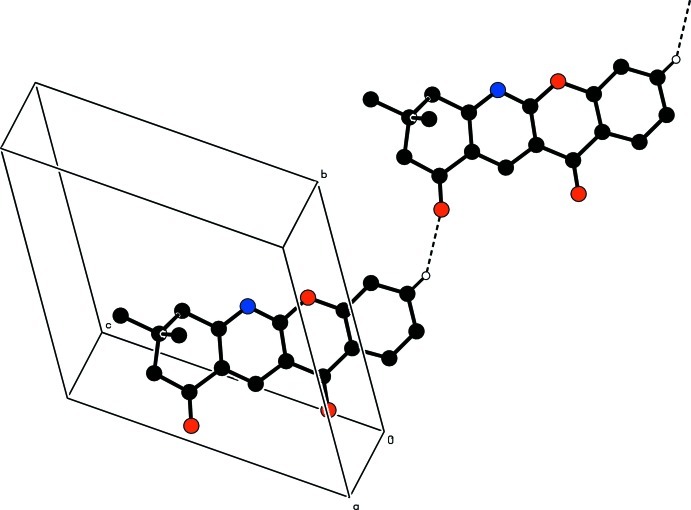
The crystal packing of the title compound viewed down a axis. H-atoms not involved in H-bonds have been excluded for clarity.

## supplementary crystallographic information

### Comment

Diketones are key intermediates in the preparation of various heterocyclic compounds (Sato *et al.*, 2008) and are popular in organic synthesis for their applications in biology and medicine. They are known to exhibit antioxidant, antitumour and antibacterial activities (Bennett *et al.*, 1999).

The bond distances and angles in the title compound (Fig. 1) agree very well with the corresponding bond distances and angles reported in a closely related compound (Öztürk Yildirim *et al.,* 2012). The dihedral angle between the pyran ring (O3/C1/C2/C10/C11/C12) and the pyridine ring (N1/C2/C3/C4/C9/C10) is 3.08 (6)°. The dihedral angle between the pyran ring and the benzene ring (C11-C16) is 2.70 (6)°. Moreover, pyridine ring makes a diheral angle of 5.78 (6)° with the benzene ring (C11-C16). The dihedral angle between the pyran ring and the cyclohexanone ring is 12.95 (6)°. In the crystal, molecules are linked *via* C15–H15···O2 hydrogen bonding interactions leading to chains running along the *b*-axis (Table 1 & Fig. 2).

### Experimental

2-Amino-4-oxo-4H-chromene-3-carbaldehyde (100 mg, 1.0 mmol) was reacted with 5,5-dimethylcyclohexane-1,3-dione (88 mg, 1.2 mmol) in the presence of yeterbium triflate (Yb(oft)3) (98 mg, 0.3 mmol) after stirring. All these reactants were dissolved in xylene (5 ml). The reaction mixture was refluxed at 398 K for 12 hours. The reaction mixture was extracted with ethyl acetate/hexane (40:60 v/v). The completion of the reaction was monitored by TLC. The end product was the title compound which was purified by column chromatography (ethyl acetate/hexane, 40:60), the yield being 80%. Single crystals suitable for X-ray diffraction were obtained by slow evaporation of a solution of the title compound in ethyl acetate at room temperature.

### Refinement

The hydrogen atoms were placed in calculated positions with C—H = 0.93 - 0.97 Å and refined in the riding model with fixed isotropic displacement parameters:*U*_iso_(H) = 1.5*U*_eq_(C) for methyl H-atoms and *U*_iso_(H) = 1.2*U*_eq_(C) for other H-atoms.

### Crystal data

**Table:**

C_18_H_15_NO_3_	*Z* = 2
*M**_r_* = 293.31	*F*(000) = 308
Triclinic, *P*1	*D*_x_ = 1.370 Mg m^−^^3^
Hall symbol: -P 1	Mo *K*α radiation, λ = 0.71073 Å
*a* = 7.4426 (6) Å	Cell parameters from 2949 reflections
*b* = 10.5117 (9) Å	θ = 2.2–26.5°
*c* = 10.6887 (9) Å	µ = 0.09 mm^−^^1^
α = 60.939 (4)°	*T* = 293 K
β = 88.107 (5)°	Block, colourless
γ = 77.546 (5)°	0.30 × 0.25 × 0.20 mm
*V* = 711.22 (10) Å^3^	

### Data collection

**Table:**

Bruker SMART APEXII area-detector diffractometer	2949 independent reflections
Radiation source: fine-focus sealed tube	2427 reflections with *I* > 2σ(*I*)
Graphite monochromator	*R*_int_ = 0.031
ω and φ scans	θ_max_ = 26.5°, θ_min_ = 2.2°
Absorption correction: multi-scan (*SADABS*; Bruker, 2008)	*h* = −9→9
*T*_min_ = 0.972, *T*_max_ = 0.982	*k* = −13→13
10655 measured reflections	*l* = −13→13

### Refinement

**Table:**

Refinement on *F*^2^	Primary atom site location: structure-invariant direct methods
Least-squares matrix: full	Secondary atom site location: difference Fourier map
*R*[*F*^2^ > 2σ(*F*^2^)] = 0.040	Hydrogen site location: inferred from neighbouring sites
*wR*(*F*^2^) = 0.118	H-atom parameters constrained
*S* = 1.05	*w* = 1/[σ^2^(*F*_o_^2^) + (0.0598*P*)^2^ + 0.1006*P*] where *P* = (*F*_o_^2^ + 2*F*_c_^2^)/3
2949 reflections	(Δ/σ)_max_ < 0.001
201 parameters	Δρ_max_ = 0.17 e Å^−^^3^
0 restraints	Δρ_min_ = −0.22 e Å^−^^3^

### Special details

**Table:**

Geometry. All esds (except the esd in the dihedral angle between two l.s. planes) are estimated using the full covariance matrix. The cell esds are taken into account individually in the estimation of esds in distances, angles and torsion angles; correlations between esds in cell parameters are only used when they are defined by crystal symmetry. An approximate (isotropic) treatment of cell esds is used for estimating esds involving l.s. planes.
Refinement. Refinement of F^2^ against ALL reflections. The weighted R-factor wR and goodness of fit S are based on F^2^, conventional R-factors R are based on F, with F set to zero for negative F^2^. The threshold expression of F^2^ > 2sigma(F^2^) is used only for calculating R-factors(gt) etc. and is not relevant to the choice of reflections for refinement. R-factors based on F^2^ are statistically about twice as large as those based on F, and R- factors based on ALL data will be even larger.

### Fractional atomic coordinates and isotropic or equivalent isotropic displacement parameters (Å^2^)

**Table:**

	*x*	*y*	*z*	*U*_iso_*/*U*_eq_	
C1	0.01482 (17)	0.15363 (14)	0.17863 (13)	0.0415 (3)	
C2	0.05259 (16)	0.17700 (13)	0.29891 (12)	0.0375 (3)	
C3	0.17358 (16)	0.07117 (13)	0.41692 (12)	0.0388 (3)	
H3	0.2332	−0.0190	0.4224	0.047*	
C4	0.20560 (16)	0.09954 (13)	0.52620 (12)	0.0382 (3)	
C5	0.33905 (17)	−0.01133 (14)	0.65130 (13)	0.0431 (3)	
C6	0.37866 (19)	0.03085 (15)	0.76134 (13)	0.0485 (3)	
H6A	0.5082	−0.0106	0.7958	0.058*	
H6B	0.3072	−0.0154	0.8423	0.058*	
C7	0.33647 (18)	0.19874 (14)	0.70916 (13)	0.0469 (3)	
C8	0.13932 (19)	0.26760 (16)	0.63542 (14)	0.0518 (3)	
H8A	0.0525	0.2290	0.7066	0.062*	
H8B	0.1136	0.3750	0.5966	0.062*	
C9	0.10985 (16)	0.23518 (13)	0.51692 (12)	0.0406 (3)	
C10	−0.03248 (16)	0.30872 (13)	0.29978 (13)	0.0387 (3)	
C11	−0.18038 (16)	0.41066 (14)	0.06841 (12)	0.0405 (3)	
C12	−0.10695 (16)	0.28199 (14)	0.05958 (13)	0.0410 (3)	
C13	−0.15013 (18)	0.28050 (16)	−0.06622 (13)	0.0488 (3)	
H13	−0.1045	0.1951	−0.0738	0.059*	
C14	−0.2593 (2)	0.40430 (17)	−0.17835 (14)	0.0542 (4)	
H14	−0.2865	0.4028	−0.2618	0.065*	
C15	−0.32914 (18)	0.53168 (16)	−0.16751 (14)	0.0523 (3)	
H15	−0.4027	0.6152	−0.2441	0.063*	
C16	−0.29078 (18)	0.53585 (15)	−0.04437 (14)	0.0481 (3)	
H16	−0.3382	0.6213	−0.0372	0.058*	
C17	0.3488 (2)	0.22292 (19)	0.83847 (16)	0.0643 (4)	
H17A	0.4726	0.1818	0.8832	0.096*	
H17B	0.2653	0.1741	0.9063	0.096*	
H17C	0.3161	0.3280	0.8070	0.096*	
C18	0.4756 (2)	0.27038 (17)	0.60480 (16)	0.0608 (4)	
H18A	0.5975	0.2276	0.6532	0.091*	
H18B	0.4458	0.3761	0.5704	0.091*	
H18C	0.4711	0.2525	0.5251	0.091*	
N1	−0.00885 (14)	0.33841 (12)	0.40449 (11)	0.0440 (3)	
O1	0.08122 (15)	0.03625 (11)	0.17871 (10)	0.0603 (3)	
O2	0.40972 (15)	−0.13281 (11)	0.66438 (11)	0.0630 (3)	
O3	−0.14882 (12)	0.42254 (9)	0.18821 (9)	0.0467 (2)	

### Atomic displacement parameters (Å^2^)

**Table:**

	*U*^11^	*U*^22^	*U*^33^	*U*^12^	*U*^13^	*U*^23^
C1	0.0459 (7)	0.0410 (7)	0.0389 (6)	−0.0114 (5)	0.0027 (5)	−0.0201 (5)
C2	0.0396 (6)	0.0378 (6)	0.0357 (6)	−0.0107 (5)	0.0034 (5)	−0.0178 (5)
C3	0.0403 (6)	0.0353 (6)	0.0399 (6)	−0.0082 (5)	0.0029 (5)	−0.0180 (5)
C4	0.0397 (6)	0.0375 (6)	0.0357 (6)	−0.0092 (5)	0.0016 (5)	−0.0164 (5)
C5	0.0449 (7)	0.0393 (7)	0.0391 (6)	−0.0088 (5)	−0.0003 (5)	−0.0149 (5)
C6	0.0535 (7)	0.0471 (7)	0.0373 (6)	−0.0078 (6)	−0.0067 (5)	−0.0157 (6)
C7	0.0558 (8)	0.0474 (7)	0.0386 (6)	−0.0082 (6)	−0.0045 (5)	−0.0229 (6)
C8	0.0595 (8)	0.0523 (8)	0.0439 (7)	0.0013 (6)	−0.0055 (6)	−0.0288 (6)
C9	0.0423 (6)	0.0415 (6)	0.0364 (6)	−0.0067 (5)	0.0011 (5)	−0.0190 (5)
C10	0.0378 (6)	0.0391 (6)	0.0365 (6)	−0.0066 (5)	0.0005 (5)	−0.0173 (5)
C11	0.0392 (6)	0.0464 (7)	0.0349 (6)	−0.0116 (5)	0.0018 (5)	−0.0186 (5)
C12	0.0429 (6)	0.0448 (7)	0.0359 (6)	−0.0150 (5)	0.0038 (5)	−0.0182 (5)
C13	0.0546 (8)	0.0542 (8)	0.0405 (7)	−0.0157 (6)	0.0023 (6)	−0.0239 (6)
C14	0.0587 (8)	0.0662 (9)	0.0370 (7)	−0.0182 (7)	−0.0020 (6)	−0.0227 (6)
C15	0.0487 (7)	0.0557 (8)	0.0383 (7)	−0.0090 (6)	−0.0044 (5)	−0.0130 (6)
C16	0.0462 (7)	0.0462 (7)	0.0431 (7)	−0.0060 (6)	−0.0022 (5)	−0.0167 (6)
C17	0.0774 (10)	0.0703 (10)	0.0496 (8)	−0.0050 (8)	−0.0112 (7)	−0.0366 (8)
C18	0.0758 (10)	0.0598 (9)	0.0538 (8)	−0.0271 (8)	0.0013 (7)	−0.0283 (7)
N1	0.0480 (6)	0.0430 (6)	0.0397 (5)	−0.0025 (5)	−0.0026 (4)	−0.0221 (5)
O1	0.0808 (7)	0.0487 (6)	0.0534 (6)	−0.0004 (5)	−0.0115 (5)	−0.0312 (5)
O2	0.0751 (7)	0.0440 (6)	0.0594 (6)	0.0067 (5)	−0.0183 (5)	−0.0241 (5)
O3	0.0520 (5)	0.0431 (5)	0.0398 (5)	0.0021 (4)	−0.0087 (4)	−0.0208 (4)

### Geometric parameters (Å, º)

**Table:**

C1—O1	1.2259 (15)	C9—N1	1.3386 (15)
C1—C12	1.4635 (17)	C10—N1	1.3256 (15)
C1—C2	1.4672 (16)	C10—O3	1.3559 (14)
C2—C3	1.3854 (16)	C11—O3	1.3778 (14)
C2—C10	1.3971 (17)	C11—C16	1.3849 (17)
C3—C4	1.3775 (16)	C11—C12	1.3924 (18)
C3—H3	0.9300	C12—C13	1.4010 (17)
C4—C9	1.4070 (17)	C13—C14	1.3723 (19)
C4—C5	1.4857 (16)	C13—H13	0.9300
C5—O2	1.2118 (15)	C14—C15	1.387 (2)
C5—C6	1.5009 (17)	C14—H14	0.9300
C6—C7	1.5299 (19)	C15—C16	1.3792 (18)
C6—H6A	0.9700	C15—H15	0.9300
C6—H6B	0.9700	C16—H16	0.9300
C7—C18	1.526 (2)	C17—H17A	0.9600
C7—C17	1.5308 (17)	C17—H17B	0.9600
C7—C8	1.5369 (18)	C17—H17C	0.9600
C8—C9	1.4963 (17)	C18—H18A	0.9600
C8—H8A	0.9700	C18—H18B	0.9600
C8—H8B	0.9700	C18—H18C	0.9600
			
O1—C1—C12	123.35 (11)	N1—C10—O3	112.77 (10)
O1—C1—C2	122.30 (11)	N1—C10—C2	125.36 (11)
C12—C1—C2	114.34 (10)	O3—C10—C2	121.86 (10)
C3—C2—C10	116.41 (11)	O3—C11—C16	115.67 (11)
C3—C2—C1	122.35 (11)	O3—C11—C12	122.82 (11)
C10—C2—C1	121.25 (11)	C16—C11—C12	121.51 (11)
C4—C3—C2	119.96 (11)	C11—C12—C13	118.32 (12)
C4—C3—H3	120.0	C11—C12—C1	120.13 (11)
C2—C3—H3	120.0	C13—C12—C1	121.55 (12)
C3—C4—C9	118.78 (10)	C14—C13—C12	120.39 (13)
C3—C4—C5	120.42 (11)	C14—C13—H13	119.8
C9—C4—C5	120.79 (10)	C12—C13—H13	119.8
O2—C5—C4	120.40 (11)	C13—C14—C15	120.18 (12)
O2—C5—C6	121.68 (11)	C13—C14—H14	119.9
C4—C5—C6	117.90 (11)	C15—C14—H14	119.9
C5—C6—C7	115.45 (10)	C16—C15—C14	120.73 (12)
C5—C6—H6A	108.4	C16—C15—H15	119.6
C7—C6—H6A	108.4	C14—C15—H15	119.6
C5—C6—H6B	108.4	C15—C16—C11	118.85 (13)
C7—C6—H6B	108.4	C15—C16—H16	120.6
H6A—C6—H6B	107.5	C11—C16—H16	120.6
C18—C7—C6	110.08 (12)	C7—C17—H17A	109.5
C18—C7—C17	109.31 (11)	C7—C17—H17B	109.5
C6—C7—C17	109.11 (11)	H17A—C17—H17B	109.5
C18—C7—C8	110.61 (11)	C7—C17—H17C	109.5
C6—C7—C8	108.18 (11)	H17A—C17—H17C	109.5
C17—C7—C8	109.53 (11)	H17B—C17—H17C	109.5
C9—C8—C7	113.02 (11)	C7—C18—H18A	109.5
C9—C8—H8A	109.0	C7—C18—H18B	109.5
C7—C8—H8A	109.0	H18A—C18—H18B	109.5
C9—C8—H8B	109.0	C7—C18—H18C	109.5
C7—C8—H8B	109.0	H18A—C18—H18C	109.5
H8A—C8—H8B	107.8	H18B—C18—H18C	109.5
N1—C9—C4	122.22 (11)	C10—N1—C9	117.23 (10)
N1—C9—C8	117.88 (11)	C10—O3—C11	119.42 (10)
C4—C9—C8	119.90 (11)		
			
O1—C1—C2—C3	3.61 (19)	C1—C2—C10—N1	178.92 (10)
C12—C1—C2—C3	−176.04 (10)	C3—C2—C10—O3	177.77 (10)
O1—C1—C2—C10	−176.52 (12)	C1—C2—C10—O3	−2.11 (18)
C12—C1—C2—C10	3.83 (16)	O3—C11—C12—C13	178.89 (10)
C10—C2—C3—C4	−0.61 (16)	C16—C11—C12—C13	−1.16 (18)
C1—C2—C3—C4	179.27 (10)	O3—C11—C12—C1	−2.24 (18)
C2—C3—C4—C9	1.67 (17)	C16—C11—C12—C1	177.71 (11)
C2—C3—C4—C5	−178.27 (10)	O1—C1—C12—C11	178.63 (12)
C3—C4—C5—O2	−6.06 (18)	C2—C1—C12—C11	−1.73 (16)
C9—C4—C5—O2	174.00 (12)	O1—C1—C12—C13	−2.54 (19)
C3—C4—C5—C6	175.48 (10)	C2—C1—C12—C13	177.10 (10)
C9—C4—C5—C6	−4.47 (17)	C11—C12—C13—C14	1.21 (19)
O2—C5—C6—C7	159.80 (13)	C1—C12—C13—C14	−177.64 (11)
C4—C5—C6—C7	−21.76 (17)	C12—C13—C14—C15	−0.5 (2)
C5—C6—C7—C18	−70.62 (14)	C13—C14—C15—C16	−0.2 (2)
C5—C6—C7—C17	169.43 (11)	C14—C15—C16—C11	0.3 (2)
C5—C6—C7—C8	50.34 (15)	O3—C11—C16—C15	−179.63 (10)
C18—C7—C8—C9	65.43 (15)	C12—C11—C16—C15	0.42 (19)
C6—C7—C8—C9	−55.20 (15)	O3—C10—N1—C9	−177.28 (10)
C17—C7—C8—C9	−174.03 (12)	C2—C10—N1—C9	1.78 (18)
C3—C4—C9—N1	−1.11 (18)	C4—C9—N1—C10	−0.57 (18)
C5—C4—C9—N1	178.84 (10)	C8—C9—N1—C10	179.47 (11)
C3—C4—C9—C8	178.85 (11)	N1—C10—O3—C11	177.09 (9)
C5—C4—C9—C8	−1.21 (17)	C2—C10—O3—C11	−2.00 (17)
C7—C8—C9—N1	−147.69 (12)	C16—C11—O3—C10	−175.71 (10)
C7—C8—C9—C4	32.35 (17)	C12—C11—O3—C10	4.23 (17)
C3—C2—C10—N1	−1.20 (18)		

### Hydrogen-bond geometry (Å, º)

**Table:**

*D*—H···*A*	*D*—H	H···*A*	*D*···*A*	*D*—H···*A*
C15—H15···O2^i^	0.93	2.43	3.250 (2)	147

Symmetry code: (i) *x*−1, *y*+1, *z*−1.
